# Editorial: The Role of Telemedicine in Transforming Healthcare Delivery—Capabilities and Barriers

**DOI:** 10.3390/healthcare13222927

**Published:** 2025-11-15

**Authors:** Motti Haimi

**Affiliations:** 1 Health Systems Management Department, The Max Stern Yezreel Valley Academic College, D.N Emek Yezreel 1930600, Israel; mottih@yvc.ac.il; 2 Rappaport Faculty of Medicine, Technion—Israel Institute of Technology, Haifa 3109601, Israel; 3 Meuhedet Healthcare Services, North District, Haifa 3109601, Israel

## 1. Introduction

The rapid evolution of digital health technologies has fundamentally reshaped healthcare delivery worldwide, with telemedicine emerging as a cornerstone of modern patient care. This transformation, dramatically accelerated by the COVID-19 pandemic, has revealed both the immense potential and persistent challenges inherent in delivering healthcare services remotely [1,2]. This Special Issue of *Healthcare* brings together a diverse collection of research and review articles that explore telemedicine’s multifaceted role in transforming healthcare delivery across various populations, clinical settings, and geographical contexts.

The ten contributions featured in this Special Issue collectively address critical dimensions of telemedicine and its implementation: from technological adoption and provider acceptance to patient satisfaction, cost-effectiveness, and equity considerations. These studies span diverse healthcare domains, including chronic disease management, mental health services, specialty care, and geriatric medicine, while examining populations ranging from socioeconomically disadvantaged urban communities to remote rural areas across multiple continents. Understanding these diverse applications is essential as telemedicine evolves from an emergency response measure to an integral component of sustainable healthcare delivery systems [3].

## 2. Provider Adoption and Acceptance

Understanding the factors that influence healthcare providers’ adoption of telemedicine is fundamental to successful implementation. Bîlbîie et al. (Contribution 1) [4] investigate the acceptance that Romanian physicians practiced for telemedicine through the Technology Acceptance Model (TAM), analyzing 1093 nationwide survey responses. Their findings reveal that perceived usefulness emerges as a stronger predictor of adoption compared to perceived ease of use, while subjective norms, perceived incentives, and accessibility to medical records significantly influence behavioral intention to use telemedicine. This study underscores that successful telemedicine implementation requires addressing not only technological barriers but also systemic factors, including regulatory frameworks and financial incentives. These findings align with broader research demonstrating that organizational support and clear reimbursement policies are critical enablers of telemedicine adoption [5,6].

The human dimension of healthcare delivery in digital settings is explored by Gabay et al. (Contribution 2) [7], who examine communication between clinicians and chronically ill elderly patients during digital encounters. Their patient-centered analysis reveals that personalized communication strategies can help preserve therapeutic relationships in virtual settings, addressing concerns about losing personal connections that are particularly acute among elderly populations. This work emphasizes that technology alone cannot ensure quality care; rather, clinicians must adapt their communication approaches to maintain empathy and connection in digital environments. The importance of the quality of communication in telemedicine has been increasingly recognized as a determinant of patient satisfaction and treatment adherence [8].

## 3. Disease-Specific Applications and Clinical Effectiveness

Several contributions demonstrate telemedicine’s efficacy in managing specific conditions and populations. Gherman et al. (Contribution 3) [9] present a prospective controlled study examining telemedicine-supported interventions versus standard care for cardiovascular risk factor management in a socially deprived urban population in Romania. Their six-month intervention, incorporating regular phone calls focused on adherence to medication, self-monitoring, and lifestyle modifications, demonstrates telemedicine’s potential to address healthcare disparities in underserved communities where traditional healthcare access remains limited. These findings contribute to growing evidence that telemedicine interventions can effectively reduce cardiovascular risk factors when integrated with patient education and remote monitoring [10,11].

Al-Aqeel et al. (Contribution 4) [12] contribute valuable evidence on the cost-effectiveness of pharmacist-led virtual diabetes clinics. Their feasibility study characterizes pharmacists’ interventions in telepharmacy settings, demonstrating improved diabetes-related outcomes while enhancing access to specialized pharmaceutical care. This work illustrates how telemedicine can expand the roles of various healthcare professionals beyond traditional boundaries, optimizing resource utilization in the management of diabetes. The integration of telepharmacy into chronic disease management represents a promising avenue for healthcare system optimization, particularly given the global shortage of healthcare professionals [13].

Haimi and Lerner (Contribution 5) [14] provide a comprehensive narrative review of telemedicine applications in celiac disease and gluten-free diet-dependent conditions, drawing insights from the COVID-19 pandemic experience. Their analysis reveals how telehealth facilitated essential dietary counseling during lockdowns and suggests sustained benefits for ongoing disease management. This review demonstrates telemedicine’s value in conditions requiring long-term nutritional support and monitoring, particularly in regions with limited access to specialized dietitians. The role of telemedicine in nutritional counseling and dietary management remains an underexplored area, warranting further investigation [15].

The application of telemedicine to specialized care is exemplified by Chen et al. (Contribution 6) [16], who assessed satisfaction with teleophthalmology services in remote areas of Taiwan. Their study validates the efficacy of an integrated real-time videoconferencing system incorporating ophthalmic instruments, demonstrating that specialty telemedicine services can effectively reach geographically isolated populations while maintaining high patient satisfaction levels. Teleophthalmology has emerged as one of the most successful applications of telemedicine, with its effectiveness demonstrated in diabetic retinopathy screening and glaucoma management [17,18].

## 4. Mental Health and Pediatric Applications

Johnson et al. (Contribution 7) [19] address a critical gap in mental health services by examining a telehealth outreach program for traumatic stress in children. Their study on Trauma-Focused Cognitive Behavioral Therapy (TF-CBT) delivery via telehealth identifies strategies for long-term sustainability, which is particularly important given documented disparities in access to mental health services for racial and ethnic minority youths and rural communities. This contribution highlights telemedicine’s potential to democratize access to evidence-based mental health interventions for vulnerable pediatric populations. The expansion of telepsychiatry and tele-mental health services was one of the most significant developments during the pandemic, with evidence suggesting comparable outcomes to in-person care for many conditions [20,21].

## 5. Health Equity and Socioeconomic Determinants

A crucial theme emerging from this Special Issue concerns health equity and the digital divide. Cobb et al. (Contribution 8) [22] present sobering findings on telehealth use among low-income African American and Latino residents of public housing in Los Angeles. Notably, their research reveals that perceived benefits and self-efficacy do not mediate the effects of demographic, health, and social determinants on telehealth utilization. These findings challenge assumptions that simply improving attitudes toward telemedicine will overcome structural barriers, emphasizing instead the need for comprehensive interventions addressing internet access, digital literacy, and systemic inequities. The persistence of digital health disparities represents one of the most pressing challenges for the implementation of telemedicine, requiring policy interventions that extend beyond healthcare systems to address broader social determinants of health [23,24].

## 6. Synthesis of Systematic Evidence 

Two comprehensive review articles provide broader perspectives on telemedicine’s evolving landscape. Bernuzzi et al. (Contribution 9) [25] offer a bibliometric analysis that maps research trends on telemedicine’s implications for healthcare professionals. Their systematic analysis of 160 articles reveals that successful telemedicine implementation requires more than technological readiness—healthcare professionals’ wellbeing must also be considered a strategic priority, as this directly impacts sustainability and quality of care. This framework reframes telemedicine as a complex organizational transformation rather than a merely technological intervention. The impact of telemedicine on healthcare workforce dynamics, including potential burnout prevention through increased flexibility and concerns about professional isolation, requires ongoing monitoring and research [26].

Pizarro-Mena et al. (Contribution 10) [27] conduct an extensive literature review on the implementation of telehealth among older people in Latin America and the Caribbean. While the COVID-19 pandemic accelerated the adoption of telehealth in gerontology and geriatrics, their analysis identifies persistent challenges, including digital literacy barriers, limited infrastructure, and cultural factors that must be addressed to ensure equitable access for aging populations in resource-limited settings. The unique needs of elderly populations in the adoption of telemedicine, including age-related sensory and cognitive changes, unfamiliarity with technology, and preference for in-person care, make tailored implementation strategies necessary [28,29].

## 7. Future Directions

Collectively, these contributions paint a nuanced picture of telemedicine’s transformation of healthcare delivery. The evidence demonstrates clear benefits: improved access to care for geographically isolated and underserved populations, enhanced chronic disease management, cost-effectiveness in specific applications, and consistent patient satisfaction across diverse clinical contexts. Telemedicine has proven particularly valuable for follow-up care, chronic disease monitoring, mental health services, and specialty consultations. However, the research also reveals significant barriers that threaten the equitable implementation of telehealth.

Infrastructure limitations, particularly inadequate broadband access in rural and low-income areas, remain fundamental obstacles [30]. The digital divide extends beyond connectivity to encompass digital literacy, device availability, and technological comfort, particularly among the elderly and socioeconomically disadvantaged populations. Provider-related barriers include concerns about reimbursement, uncertainty regarding regulation, and the need for training in telemedicine-specific communication skills [31]. System-level challenges involve interoperability, data security, and the integration of telemedicine platforms with existing healthcare infrastructure.

## 8. Critical Research Gaps and Future Priorities

While this Special Issue advances our understanding of telemedicine’s implementation, several critical research gaps warrant attention. First, longitudinal outcome studies are needed to assess the long-term effectiveness of telemedicine interventions across various clinical conditions. Most existing studies, including several in this collection, focus on short-to-medium term outcomes. Research examining 5–10-year outcomes, including clinical endpoints, healthcare utilization patterns, and patient satisfaction trajectories, would provide crucial evidence for sustainable telemedicine integration [32].

Second, comparative effectiveness research must expand beyond simple telemedicine-versus-usual-care designs to examine optimal modalities for specific conditions and populations. Which patients benefit most from synchronous video consultations versus asynchronous store-and-forward approaches? When are hybrid models superior to purely virtual or in-person care? Understanding these nuances will enable precise implementation tailored to patient needs and healthcare contexts [33].

Third, the economic evaluation of telemedicine remains incomplete. While studies like Al-Aqeel et al. [12] provide valuable feasibility data, comprehensive health economic analyses incorporating societal costs, productivity gains, patient time savings, and long-term cost trajectories are essential for policy decision-making. Standardized frameworks for telemedicine’s cost-effectiveness assessment across different healthcare systems would facilitate evidence synthesis and policy translation [34].

Fourth, the implementation of science research must identify effective strategies for scaling telemedicine in diverse settings. The contributions by Johnson et al. [19] and Pizarro-Mena et al. [27] highlight sustainability challenges, but more research is needed on organizational factors, workflow integration, payment models, and training programs that enable successful large-scale implementation. Comparative implementation studies across different healthcare systems could identify transferable success factors [35].

Fifth, research on the integration of artificial intelligence with telemedicine represents an area for development. While not extensively covered in this Special Issue, AI-assisted triage, diagnostic support, and automated monitoring have the potential to enhance telemedicine capabilities while raising new questions about clinical validation, liability, and the preservation of human connection in care delivery [36,37]. Studies examining optimal human–AI collaboration in telemedicine settings are urgently needed.

Sixth, research on patient preference and satisfaction must move beyond simple satisfaction scores to understand the factors driving patient choices between telemedicine and in-person care. What clinical scenarios do patients prefer to receive through virtual care? How do preferences vary by demographic characteristics, health literacy, prior technology experience, and clinical condition? Understanding patient decision-making will inform the design of patient-centered hybrid care models [38].

Finally, health equity research must examine not only disparities in access but also disparities in quality for the delivery of care through telemedicine. Do outcomes differ by race, ethnicity, socioeconomic status, or language proficiency when care is delivered via telemedicine? Are certain populations receiving lower-quality virtual care due to communication barriers, technological limitations, or implicit biases? As Cobb et al. [22] demonstrate, structural interventions beyond changes in attitude are necessary to achieve health equity in digital healthcare delivery [39,40].

## 9. Implications for Policy and Practice 

Moving forward, several priority areas emerge from this body of work. First, policies must address structural barriers to access, including universal broadband infrastructure, device provision programs, and digital literacy initiatives targeted at vulnerable populations. Telemedicine cannot achieve its potential for equity without fundamental investments in digital infrastructure as a social determinant of health [41,42].

Second, reimbursement models must evolve to fairly compensate telemedicine services while incentivizing quality care. Payment parity between virtual and in-person visits, when clinically appropriate, should be accompanied by quality metrics ensuring that telemedicine maintains high standards of care delivery. Value-based payment models may better capture telemedicine’s contributions to access, convenience, and prevention than traditional fee-for-service approaches [43].

Third, provider training programs should incorporate telemedicine-specific competencies, including digital communication skills, cultural sensitivity in virtual care delivery, and proficiency with various telemedicine platforms and technologies. Medical education must evolve to prepare future healthcare professionals for hybrid practice environments where telemedicine is integrated seamlessly with in-person care [44].

Fourth, regulatory frameworks must balance innovation with patient safety. Licensure portability, privacy protections, prescribing regulations, and liability standards need modernization to support telemedicine expansion while safeguarding patients. International collaboration on telemedicine standards and regulations could facilitate cross-border care delivery where appropriate [45].

## 10. Conclusions

The pandemic served as an unprecedented catalyst for the adoption of telemedicine, demonstrating both its potential and its limitations. As healthcare systems transition to post-pandemic normalcy, the challenge lies in preserving telemedicine’s benefits while addressing its shortcomings. Hybrid care models that thoughtfully integrate virtual and in-person services may offer the most promising path forward, allowing personalized care delivery that leverages technology’s strengths while maintaining essential aspects of traditional healthcare relationships.

This Special Issue contributes valuable evidence to guide the continued evolution of telemedicine. By examining its implementation across diverse populations, healthcare systems, and clinical applications, these studies provide actionable insights for policymakers, healthcare administrators, clinicians, and researchers working to ensure that telemedicine fulfills its promise of becoming more accessible, efficient, and equitable healthcare for all. The research gaps identified here represent opportunities for the scientific community to advance knowledge and improve practice, ultimately enhancing patient care in an increasingly digital healthcare landscape.
